# In Vitro Activity of Silver-Bound Titanium Dioxide (TiAB) Against Multidrug-Resistant Vaginal Pathogens

**DOI:** 10.3390/diseases13110366

**Published:** 2025-11-10

**Authors:** Lorenzo Drago, Luigi Regenburgh De La Motte, Erika Stefàno, Vincenzo Minasi, Loredana Deflorio, Sofia Benedetti, Fabiana Giarritiello

**Affiliations:** 1 UOC Laboratory of Clinical Medicine with Specialized Areas, IRCCS MultiMedica, 20138 Milan, Italy; lorenzo.drago@unimi.it (L.D.); luigi.delamotte@multimedica.it (L.R.D.L.M.); erika.stefano@multimedica.it (E.S.); vincenzo.minasi@multimedica.it (V.M.); loredana.deflorio@multimedica.it (L.D.); sofia.benedetti@multimedica.it (S.B.); 2 Clinical Microbiology and Microbiome Laboratory, Department of Biomedical Sciences for Health, University of Milan, 20133 Milan, Italy; 3 Department of Medicine and Health Sciences “V. Tiberio”, University of Molise, 86100 Campobasso, Italy

**Keywords:** TiAB, titanium dioxide, silver ions, vaginal infections, antimicrobial activity, MIC, MBC, time-kill assay, clinical isolates, gynecological pathogens

## Abstract

**Background:** Gynecological infections, including bacterial vaginosis, vulvovaginal candidiasis, and recurrent urinary tract infections, represent a major clinical burden and are often complicated by biofilm formation and antimicrobial resistance. Novel non-antibiotic strategies are urgently needed. We previously demonstrated the antimicrobial activity of silver-bound titanium dioxide (TiAB) against multidrug-resistant bacteria isolated from dermatological infections. **Objectives:** We evaluated whether TiAB, at concentrations used in marketed medical devices, exerts antibacterial and antifungal effects against clinically relevant vaginal isolates by determining Minimum Inhibitory Concentration/ Minimum Bactericidal and Fungicidal Concentration (MIC, MBC/MFC), and time–kill kinetics. **Methods:** A total of 73 clinical isolates were collected from vaginal swabs, including Staphylococcus aureus (MSSA, MRSA), *Escherichia coli* (ESBL+ and non-ESBL), *Klebsiella pneumoniae*, *Enterococcus* spp., *Streptococcus agalactiae*, and *Candida albicans*. Minimum inhibitory concentrations (MICs) and minimum bactericidal/fungicidal concentrations (MBCs/MFCs) were determined by broth microdilution, and bactericidal activity was confirmed by time-kill assays. **Results:** TiAB exhibited potent activity against Gram-negative bacteria, with median MIC values of 1–2% (*w*/*v*) for *E. coli* and *K. pneumoniae*. Gram-positive isolates, including *S. agalactiae* and *Enterococcus* spp., showed higher MIC values (2–4%). Candida albicans displayed fungistatic inhibition at 4%. Time-kill assays confirmed rapid bactericidal effects for Gram-negative isolates within 8 h at 2× MIC, while Gram-positive bacteria required prolonged exposure. **Conclusions:** These findings extend previous evidence of TiAB’s antimicrobial properties to gynecological pathogens, supporting its potential as a topical, non-antibiotic option for managing vaginal infections in an era of rising antimicrobial resistance. Further in vivo validation is warranted.

## 1. Introduction

Vaginal infections are among the most frequent gynecological conditions worldwide, representing a major burden for women’s health across all ages. They typically arise from alterations in the vaginal microbiota, where the loss of protective *Lactobacillus* spp. facilitates the overgrowth of pathogenic bacteria or fungi. This dysbiosis leads not only to local symptoms such as abnormal discharge, irritation, and discomfort but also increases susceptibility to recurrent and secondary infections, with significant clinical and social consequences [1,2,3]. One of the most common conditions is bacterial vaginosis (BV), strongly associated with *Gardnerella vaginalis*. This microorganism contributes to biofilm formation and immune evasion, mechanisms that complicate eradication and explain the high recurrence rates despite antibiotic treatment [3]. Another clinically relevant pathogen is *Escherichia coli*, which, although primarily recognized as a cause of urinary tract infections, can also colonize the vaginal tract, particularly in women with recurrent UTIs, acting as a reservoir for ascending infections [4]. In addition, *Streptococcus agalactiae* (Group B *Streptococcus*, GBS)is a major vaginal colonizer of concern during pregnancy, as vertical transmission can result in severe neonatal infections, preterm birth, and maternal complications [5,6]. Fungal pathogens such as *Candida albicans* and *Candida glabrata* are responsible for recurrent vulvovaginal candidiasis, a condition affecting up to 5–8% of women and increasingly difficult to treat due to emerging resistance to azole antifungals [7]. Conventional therapies include antibiotics (e.g., metronidazole, clindamycin) for BV and antifungal agents (e.g., fluconazole) for candidiasis. However, the growing problem of antimicrobial resistance (AMR) has been accompanied by treatment failures and frequent recurrences [8]. Probiotic supplementation has been explored as an adjunctive approach to restore *lactobacilli* and reduce recurrences, but results remain heterogeneous and inconclusive [9]. Given these limitations, there is a pressing need for novel non-antibiotic antimicrobial strategies specifically tailored to gynecological infections. Silver-bound titanium dioxide (TiAB) is an innovative compound with sustained silver-ion release and demonstrated antibacterial and antibiofilm activity in dermatological and surgical settings [10,11,12,13,14,15,16]. However, its efficacy against vaginal pathogens has not been previously explored. This study therefore evaluates, for the first time, the in vitro antimicrobial activity of TiAB against a comprehensive panel of clinically relevant vaginal bacteria and fungi, using MIC, MBC/MFC, and time-kill assays, with the aim of assessing its potential role as a topical gynecological antimicrobial agent. Unlike our dermatology-focused investigation, the present work exclusively addresses vaginal clinical isolates and their susceptibility profiles, thereby minimizing textual and dataset overlap while extending TiAB evaluation to a distinct clinical niche.

## 2. Materials and Methods

### 2.1. Clinical Isolates

A total of 73 clinical isolates were included in this study. Strains were collected at the IRCCS MultiMedica Laboratory (Milan, Italy) as part of routine microbiological diagnostics and subsequently archived in the institutional microbial strain library at −80 °C using the microvial cryopreservation system. The isolates originated from vaginal swabs, urine samples, and tissue biopsies of women presenting with symptomatic infections. Identification at the time of isolation was performed with standard biochemical methods using the VITEK^®^ automated system (bioMérieux, Marcy-l’Étoile, France), upon thawing, all isolates were reconfirmed standard biochemical profiling and selective media. The collection consisted of 15 *Streptococcus agalactiae* (Group B *Streptococcus*, GBS), 5 *Gardnerella vaginalis*, 3 *Neisseria gonorrhoeae*, 15 *Escherichia coli*, 15 *Enterococcus* spp. (predominantly *E. faecalis* and *E. faecium*), 15 *Candida albicans*, and 5 *Candida glabrata*. Prior to antimicrobial testing, each isolate was thawed and subcultured on selective agar media to ensure viability. *G. vaginalis* was cultured on Columbia Agar with 5% Sheep Blood (COS agar, bioMérieux, code 43041) under a 5% CO_2_ atmosphere. *N. gonorrhoeae* was maintained on PVX Chocolate Agar with PolyViteX™ supplement (bioMérieux, code 43611) under identical incubation conditions. Fungal strains (*C. albicans* and *C. glabrata*) were plated on Sabouraud Gentamicin Chloramphenicol 2 Agar (SGC2, bioMérieux, code 43651). *S. agalactiae*, *E. coli*, and *Enterococcus* spp. were routinely cultured on COS agar at 37 °C under aerobic conditions. After 24 h incubation, inocula were prepared by suspending colonies in Brain Heart Infusion (BHI) broth (bioMérieux, code 42081) to a 0.5 McFarland standard (approximately 1.5 × 10^8^ CFU/mL), measured with a DensiCHECK Plus spectrophotometer (bioMérieux). This inoculum density was deliberately chosen to simulate the high microbial burden typically associated with vaginal infections [17].

### 2.2. Preparation of TiAB Suspension

TiAB powder was kindly provided by Eurokemical S.r.l. (Covo, Italy). The compound consisted of 99.10% titanium dioxide (TiO_2_) with covalently bound silver ions (Ag^+^) in a concentration range of 0.18–0.23%. Sterility was confirmed by a microbial count < 100 CFU/g. A stock suspension (16% *w*/*v*) was prepared by dissolving 2 g of TiAB in 12.5 mL of BHI broth. The product forms a microcrystalline, water-dispersible suspension designed for sustained silver-ion (Ag^+^) release rather than bulk dissolution; insoluble TiO_2_ microcrystals remain as particulate matter during testing. According to the manufacturer’s specifications for this batch, the pH of a 2% aqueous dispersion is ~3.5, the moisture content is ~0.1%, and the total microbial count < 100 CFU/g; particle size is specified as microcrystalline (non-nanoscale), consistent with stable suspensions used in topical medical devices. (No claim is made on bond strength quantitation.). To maintain uniform dispersion and prevent sedimentation, the suspension was vortexed thoroughly, continuously agitated during experimental procedures, and stored under sterile conditions at room temperature until use.

### 2.3. MIC and MBC/MFC Determination

The minimum inhibitory concentration (MIC) and minimum bactericidal/fungicidal concentration (MBC/MFC) were determined following the Clinical and Laboratory Standards Institute (CLSI) guidelines—M100 for bacteria and M27 for yeasts. Experimental procedures were performed in accordance with our previously published study on dermatological isolates [10], adapted here for gynecological pathogens to ensure methodological comparability. Serial two-fold dilutions of TiAB (ranging from 8% to 0.01% *w*/*v*) were prepared in 96-well microtiter plates. Each well contained 100 μL of TiAB suspension, 100 μL of broth, and 100 μL of microbial inoculum (0.5 McFarland). For *Candida* spp., Sabouraud broth was used in place of BHI. Plates were incubated at 35 ± 2 °C with continuous agitation to minimize sedimentation. MIC *was* defined as the lowest concentration at which no visible microbial growth was observed after 24 h. To standardize pipetting from a particulate suspension, plates were gently vortexed (5 s) immediately prior to dispensing; aliquots were withdrawn from the homogenized supernatant with wide-bore tips, and endpoint reads were performed above the sedimented layer. Subculture confirmation minimized visual bias. A subset of isolates was also validated by parallel plate-counting, confirming concordance between visual and CFU-based determinations. For MBC/MFC evaluation, 10 μL aliquots from the MIC well and three preceding dilutions were plated on the appropriate agar medium used for primary subculture (COS agar for *E. coli*, *Enterococcus* spp., *S. agalactiae* and *G. vaginalis*; PVX Chocolate agar with PolyViteX™ for *N. gonorrhoeae*; SGC2 agar for *Candida* spp.). The MBC/MFC was defined as the lowest TiAB concentration producing a ≥99.9% reduction in CFU compared with the initial inoculum. Given the discrete and non-normally distributed nature of MIC/MBC values, data were expressed as medians rather than means. Because TiAB is a particulate suspension rather than a true solution, concentrations are reported as % (*w*/*v*), which more accurately reflect experimental exposure than converting to µg/mL for insoluble metal–oxide composites. Representative photographic documentation of turbidity and sediment handling has been provided as Figure S1 in the Supplementary Materials.

### 2.4. Time-Kill Assays

Time-kill kinetics were performed to investigate bactericidal and fungicidal dynamics. Strains were incubated in BHI (or Sabouraud broth for *Candida* spp.) at 0.5 McFarland in the presence of TiAB at 0.5×, 1×, and 2× MIC. Cultures were maintained at 37 °C in a mechanical shaker-incubator to ensure homogeneous exposure.

Aliquots were withdrawn at 0, 2, 4, 6, 8, and 24 h, serially diluted, and plated on selective agar. CFU/mL were enumerated after incubation. A ≥3 log_10_ CFU/mL reduction from baseline was considered bactericidal, while reductions < 3 log_10_ were considered bacteriostatic. All assays were performed in duplicate. Median values were reported to represent central trends while limiting the impact of outliers.

### 2.5. Statistical Analysis

MIC and MBC/MFC results were summarized using median values due to the non-parametric distribution of the data. Differences between Gram-positive and Gram-negative bacterial groups were evaluated using the Mann–Whitney U test. Fungal isolates were analyzed descriptively. Time-kill data were expressed as CFU/mL over time and reported as medians of duplicate experiments. Statistical analysis was conducted with MedCalc Statistical Software version 20.218 (MedCalc Software Ltd., Ostend, Belgium; https://www.medcalc.org, accessed on 24 September 2025) A *p*-value < 0.05 was considered statistically significant.

## 3. Results

### 3.1. MIC and MBC/MFC

The in vitro antimicrobial activity of TiAB was evaluated against 73 clinical isolates associated with vaginal infections. The full dataset, including values for individual strains, is available in Supplementary Table S1, inter–strain variability was considerable for several species, which justified reporting medians and underscores the need for larger isolate panels in follow-up studies. A summary of the aggregated results is presented in Table 1. TiAB demonstrated consistent antimicrobial activity across the tested strains.

Median MIC values ranged from 1% to 4%. The most susceptible organisms were *G. vaginalis* and *C. albicans* (median MIC: 1%). *N. gonorrhoeae* showed intermediate susceptibility (2%), while *S. agalactiae* and *C. glabrata* had the highest MIC values (4%). MBC/MFC values ranged from 1% to 8%. *G. vaginalis* displayed the lowest MBC (1%), identical to its MIC, indicating rapid bactericidal activity. *C. glabrata* followed with an MFC of 4%. In contrast, *S. agalactiae* and *Enterococcus* spp. required the highest concentrations (8%) to achieve microbial clearance. *C. albicans* showed a median MBC of 4%, two dilutions above its MIC, while *N. gonorrhoeae* had a moderate MBC of 3%. These findings suggest that TiAB exerts a primarily bactericidal or fungicidal effect, particularly against Gram-negative bacteria (*G. vaginalis* and *N. gonorrhoeae*) and *Candida* species. However, Gram-positive organisms (GBS and *Enterococcus* spp.) may require higher concentrations or longer exposure for complete eradication [18,19].

#### Statistical Results

**Figure 1:**
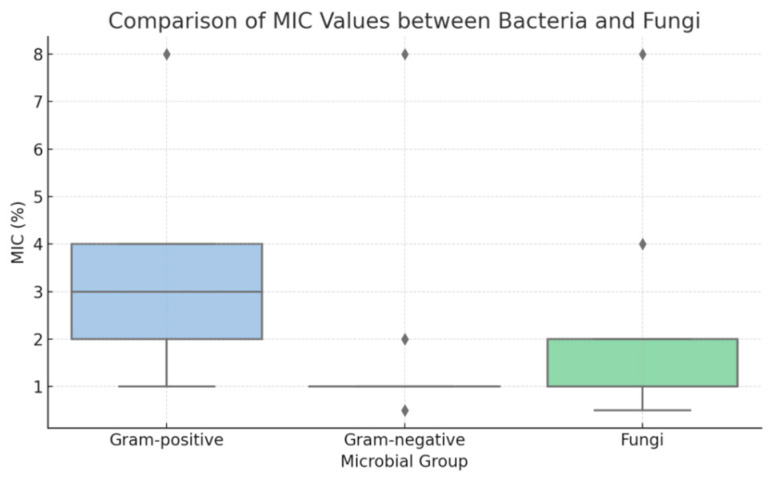
Boxplot of minimum inhibitory concentration (MIC) values for Gram-positive bacteria, Gram-negative bacteria, and fungal isolates (*Candida* spp.). Gram-negative bacteria and fungi displayed significantly lower MIC distributions compared to Gram-positive bacteria, indicating higher susceptibility to TiAB.

To further explore susceptibility patterns, statistical analysis was performed on MIC and MBC values to compare Gram-positive and Gram-negative bacteria. Due to the discrete and non-parametric nature of the data, results were summarized using median values, and comparisons were conducted using the Mann–Whitney U test. The analysis revealed that Gram-negative bacteria (e.g., *G. vaginalis*, *N. gonorrhoeae*) exhibited significantly lower MIC values than Gram-positive species (*Enterococcus* spp., *S. agalactiae*) (*p* < 0.001). This trend was also evident in MBC values, confirming a greater susceptibility of Gram-negative organisms to TiAB. Fungal isolates (*C. albicans* and *C. glabrata*) clustered more closely with Gram-negative bacteria in terms of MIC distribution. These differences are illustrated in the boxplot presented in Figure 1.

### 3.2. Time-Kill Assay Results

**Figure 2:**
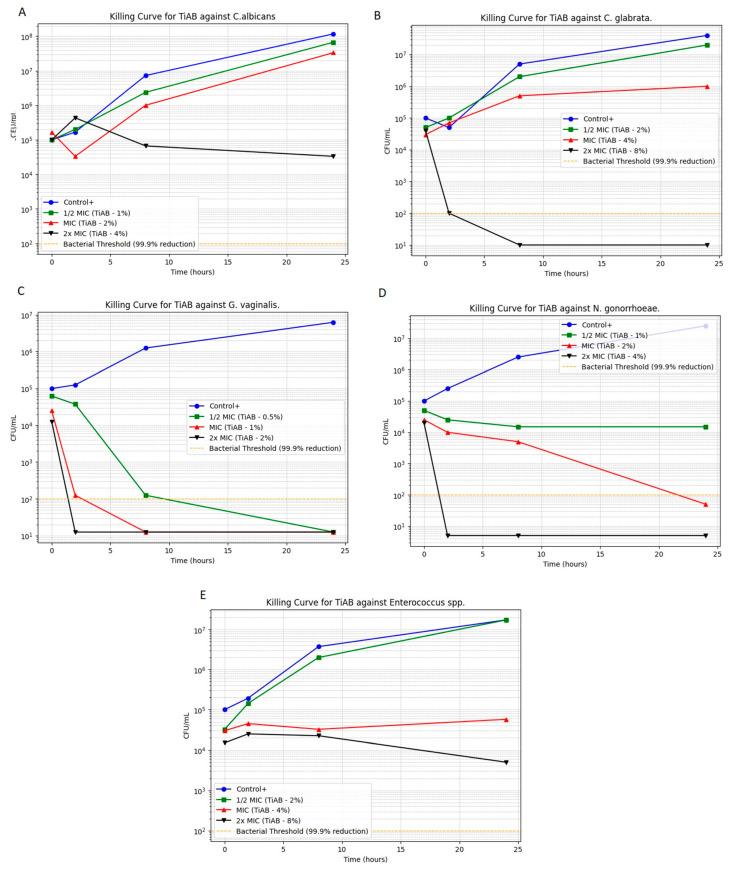
Time-kill curves for selected vaginal pathogens exposed to TiAB at 0.5×, 1×, and 2× MIC. Panels show: (**A**) *Candida albicans*; (**B**) *Candida glabrata*; (**C**) *Gardnerella vaginalis*; (**D**) *Neisseria gonorrhoeae*; (**E**) *Enterococcus* spp. Results for *Escherichia coli* are shown on a previously published data [10], which confirmed similar killing dynamics.

Time-kill assays were performed to evaluate the bactericidal and fungicidal activity of TiAB over time against selected vaginal pathogens. The microbial growth kinetics at 0.5×, 1×, and 2× MICs are illustrated in Figure 2 (panels A–E), highlighting the time- and concentration-dependent effects of TiAB. Among Gram-negative bacteria, TiAB exhibited rapid bactericidal activity. *N. gonorrhoeae* (panel D) demonstrated substantial reduction at 2% and complete killing at 4% within 8 h. Similarly, *G. vaginalis* (panel C) displayed a 3-log_10_ CFU/mL reduction within 6 h at 1%, with total eradication at 2%. Results for *E. coli* were consistent with those previously work given the overlap in isolates, detailed data are detailed data are published elsewhere [10], but overall findings confirmed the reproducible activity of TiAB against this pathogen across distinct clinical contexts.

In contrast, Gram-positive bacteria responded more slowly. *Enterococcus* spp. (panel E) exhibited a primarily bacteriostatic effect at 4%, with gradual CFU reduction and partial bactericidal activity at 8% over 24 h. *S. agalactiae* was excluded from this assay due to MIC and MBC values exceeding the testable concentration range (>8%). Regarding fungal pathogens, *C. albicans* exhibited lower MIC/MFC thresholds (1%/4%) and a concentration-dependent reduction, whereas *C. glabrata* required higher exposure (4%) and showed delayed killing, consistent with its reduced susceptibility relative to *C. albicans*. Gram-negative species such as *Escherichia coli* and *Klebsiella pneumoniae* exhibited MIC and MBC values of 1% and 2% (*w*/*v*), respectively, comparable to those observed for *Enterococcus faecalis*, indicating that TiAB maintains inhibitory activity across both Gram-positive and Gram-negative vaginal isolates, similarly to in our previous work.

## 4. Discussion

This study evaluated the in vitro antimicrobial activity of TiAB, a silver–titanium compound, against a panel of pathogens commonly involved in vaginal infections. The experimental design focused exclusively on TiAB without including reference antibiotics or antifungals, since its mechanism of action and intended applications differ from those of conventional drugs. Comparative testing with other silver- or metal-based biomaterials was not feasible at this stage, but future work will include assays with AgNO_3_ and plain TiO_2_ to clarify the individual contribution of silver ions and the TiO_2_ matrix. TiAB demonstrated broad-spectrum efficacy across clinically relevant vaginal pathogens. The strongest activity was observed against *G. vaginalis* and *N. gonorrhoeae*, both showing low MIC/MBC values and rapid eradication in time-kill assays. Among fungal pathogens, *C. glabrata* was more susceptible than *C. albicans*, with near-complete clearance at 2× MIC within 24 h, while *C. albicans* exhibited a slower, concentration-dependent reduction. These results align with the known variability in antifungal susceptibility among *Candida* species and highlight the potential role of TiAB in recurrent or resistant vulvovaginal candidiasis. TiAB combines contact-mediated membrane damage by TiO_2_-bound silver with a controlled Ag^+^ release from the microcrystalline surface, which together account for the rapid killing observed in Gram-negatives and the slower, exposure-dependent effect in Gram-positives. This differential response is consistent with previous reports on silver-based antimicrobials [20,21,22,23,24] and may be explained by structural differences: Gram-negative bacteria possess thinner peptidoglycan layers and more permeable outer membranes, facilitating silver ion penetration, while Gram-positive organisms such as *S. agalactiae* and *Enterococcus* spp. have thicker cell walls that may delay bactericidal activity [25,26,27,28]. For *E. coli*, the findings were consistent with our previously published work in a different clinical setting, confirming the reproducible antimicrobial activity of TiAB across distinct infection sites [10]. To avoid redundancy, detailed *E. coli* results are not presented here. From a translational standpoint, these data support the potential of TiAB in gynecological formulations such as creams, ovules, or gels, where direct topical application could help reduce pathogen load and biofilm-associated infections. This is particularly relevant for bacterial vaginosis, gonococcal infections, and recurrent candidiasis, which are often difficult to manage due to high recurrence rates and increasing antimicrobial resistance. However, several limitations must be acknowledged. First, this study focused solely on antimicrobial efficacy and did not assess cytotoxicity or compatibility with vaginal epithelial tissues. These aspects are essential before advancing toward clinical use. Second, in vivo studies will be required to evaluate safety, tolerability, and long-term effectiveness in the gynecological setting. Moreover, antimicrobial susceptibility testing was performed under standard neutral pH conditions, following CLSI guidelines to ensure reproducibility and comparability with other studies. While future work will evaluate TiAB activity at acidic pH (4.0–4.5) to better simulate the vaginal microenvironment, maintaining neutral conditions in the present study was necessary to guarantee standardization and to support the growth of *Candida species*, which are markedly inhibited under acidic conditions. This approach allowed a reliable assessment of TiAB’s intrinsic antimicrobial potential rather than its behavior in the vaginal milieu. Finally, direct comparisons with existing topical antimicrobials would provide additional context for positioning TiAB as a therapeutic option. As biofilm formation is clinically relevant and the vaginal environment is acidic and rich in lactate, future assays will evaluate TiAB activity under simulated vaginal conditions. Moreover, future experiments will investigate the antibiofilm activity of TiAB, particularly against *G. vaginalis*, to extend these preliminary findings from planktonic to sessile bacterial forms.

## 5. Conclusions

This study provides the first evidence that TiAB exhibits effective in vitro antimicrobial activity against multidrug-resistant vaginal isolates, including *G. vaginalis*, *E. faecalis*, *S. aureus*, *E. coli*, and *C. albicans*. The observed MIC and MBC/MFC values ranged between 1 and 4% (*w*/*v*), confirming that TiAB, at concentrations already used in existing medical devices, can inhibit both bacterial and fungal growth without the addition of conventional antibiotics. The present work focused on planktonic forms to evaluate the intrinsic antimicrobial potential of TiAB according to standardized protocols, while future studies will assess its antibiofilm activity, cytocompatibility on vaginal epithelial cells, and performance under acidic (pH 4.0–4.5) conditions typical of the vaginal environment. Overall, these results provide a solid rationale for further preclinical evaluation of TiAB-containing formulations as potential candidates for the management of recurrent or resistant vaginal infections.

## Figures and Tables

**Table 1:** Median MIC and MBC/MFC values of TiAB against clinically relevant vaginal pathogens.

Pathogen	N°. of Isolates	MIC Median (%)	MBC/MFC Median (%)
*Streptococcus agalactiae (GBS)*	15	4.0	8.0
*Gardnerella vaginalis*	5	1.0	1.0
*Neisseria gonorrhoeae*	3	2.0	3.0
*Enterococcus* spp.	15	2.0	8.0
*Candida albicans*	15	1.0	4.0
*Candida glabrata*	5	4.0	4.0

Values represent the median minimum inhibitory concentration (MIC) and minimum bactericidal/fungicidal concentration (MBC/MFC) expressed as percentages (%) for each group of clinical isolates tested. Median values were used to better represent the central tendency of susceptibility data and to minimize the influence of outliers. MIC values represent the lowest TiAB concentrations that inhibited visible microbial growth. MBC/MFC values indicate the concentrations required for ≥99.9% microbial reduction.

## Data Availability

The data that support the findings of this study are not openly available due to reasons of sensitivity and are available from the corresponding author upon reasonable request. Data are stored in a controlled-access repository at IRCCS MultiMedica (Milan, Italy).

## Supplementary Materials

The following supporting information can be downloaded at: https://www.mdpi.com/article/10.3390/diseases13110366/s1. Table S1: Minimum inhibitory concentrations (MICs) and minimum bactericidal/fungicidal. concentrations (MBCs/MFCs) of TiAB against 73 clinical vaginal isolates, determined by broth microdilution assays. Values are expressed as percentages of TiAB. Abbreviations: MIC, minimum inhibitory concentration; MBC, minimum bactericidal concentration; MFC, minimum fungicidal concentration. Figure S1: Representative photographic documentation of turbidity and sediment handling.

## Abbreviations

MIC	Minimum Inhibitory Concentration
MBC	Minimum Bactericidal Concentration
CFU	Colony Forming Unit
TiAB	Titanium dioxide microcrystals with silver ions
CLSI	Clinical and Laboratory Standards Institute
