# Supplementary material for: In Vitro Activity of Silver-Bound Titanium Dioxide (TiAB) Against Multidrug-Resistant Vaginal Pathogens
Source: Diseases. 2025 Nov 10;13(11):366. doi: 10.3390/diseases13110366 (PMC12651475; doi:10.3390/diseases13110366)
Supplement: Supplementary file 1 [file diseases-13-00366-s001.zip › diseases-3932791-supplementary.pdf]

SUPPLEMENTARY MATERIALS

Supplementary Table S1. Minimum inhibitory concentrations (MIC) and minimum bactericidal/fungicidal concentrations (MBC/MFC) of TiAB against 73 clinical vaginal isolates, determined by broth microdilution assays. Values are expressed as percentages of TiAB. Abbreviations: MIC, minimum inhibitory concentration; MBC, minimum bactericidal concentration; MFC, minimum fungicidal concentration

Pathogen	Strain	MIC	Median MIC value	MBC	Median MBC value
Streptococcus agalactiae	1	4%	**4.0 %**	n/a	8.0 %
	2	8%		n/a	
	3	2%		8%	
	4	1%		8%	
	5	4%		n/a	
	6	4%		n/a	
	7	8%		n/a	
	8	4%		n/a	
	9	2%		n/a	
	10	2%		n/a	
	11	2%		8%	
	12	4%		n/a	
	13	4%		n/a	
	14	2%		n/a	
	15	1%		8%	
Gardnerella vaginalis	1	1%	**1.0 %**	1%	**1.0 %**
	2	1%		2%	
	3	1%		1%	
	4	1%		1%	
	5	1%		1%	
Neisseria gonorrhoeae	1	8%	**2.0 %**	n/a	**3.0 %**
	2	1%		2%	
	3	2%		4%	
Escherichia coli	1	1%	**1.0 %**	4%	2.0 %
	2	1%		4%	
	3	0,5%		1%	
	4	1%		2%	
	5	2%		2%	
	6	1%		2%	
	7	0,5%		4%	
	8	1%		4%	
	9	1%		2%	
	10	2%		4%	
	11	0,5%		1%	
	12	1%		2%	
	13	1%		2%	
	14	2%		4%	
	15	1%		2%	
Enterococcus spp.	1	4%	**2.0 %**	n/a	**8.0 %**

	2	4%		8%	
	3	2%		8%	
	4	4%		8%	
	5	2%		8%	
	6	4%		8%	
	7	4%		8%	
	8	2%		4%	
	9	4%		8%	
	10	2%		8%	
	11	1%		4%	
	12	1%		8%	
	13	2%		4%	
	14	4%		8%	
	15	1%		4%	
Candida albicans	1	1%	**1.0 %**	4%	**4.0 %**
	2	0,5%		4%	
	3	2%		n/a	
	4	0,5%		8%	
	5	1%		4%	
	6	2%		n/a	
	7	2%		n/a	
	8	1%		4%	
	9	1%		4%	
	10	0,5%		4%	
	11	0,5%		2%	
	12	1%		4%	
	13	1%		4%	
	14	2%		n/a	
	15	1%		2%	
Candida glabrata	1	4%	**4.0 %**	4%	**4.0 %**
	2	2%		2%	
	3	2%		4%	
	4	4%		4%	
	5	8%		8%	

Complete MIC and MBC/MFC values of TiAB for all 73 clinical isolates tested in this study. Each row reports the MIC and MBC/MFC values for a single strain, grouped by pathogen species. On the right, the median MIC and MBC/MFC values for each species are expressed as percentages (%). These data provide a detailed overview of the inter-strain variability in TiAB susceptibility among gynecological pathogens.

Supplementary Figure

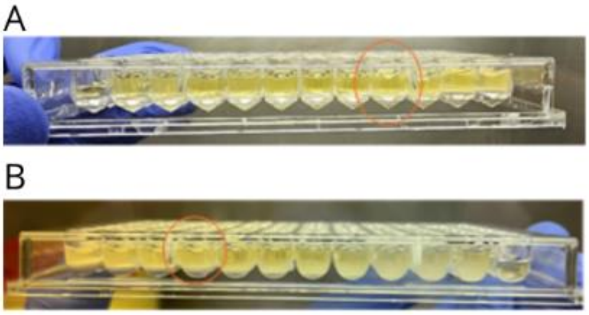

Figure S1. The panel A shows *Gardnerella vaginalis* and panel B *Candida albicans* in the MIC assay. The MIC concentrations are highlighted (orange circle), representing the first well in which the BHI solution above the sedimented TiAB appears clear, comparable to the negative control, with no turbidity indicating microbial inhibition.
